# Downregulation of miR-146b-3p Inhibits Proliferation and Migration and Modulates the Expression and Location of Sodium/Iodide Symporter in Dedifferentiated Thyroid Cancer by Potentially Targeting MUC20

**DOI:** 10.3389/fonc.2020.566365

**Published:** 2021-01-08

**Authors:** Shasha Hou, Xiaorui Xie, Jing Zhao, Cailan Wu, Ning Li, Zhaowei Meng, Chunquan Cai, Jian Tan

**Affiliations:** ^1^ Department of Nuclear Medicine, Tianjin Medical University General Hospital, Tianjin, China; ^2^ Department of Pediatrics, Tianjin Medical University General Hospital, Tianjin, China; ^3^ Department of Ultrasound, Tianjin Medical University Cancer Institute and Hospital, Tianjin, China; ^4^ Department of Nuclear Medicine, Tianjin Fourth Central Hospital, Tianjin, China; ^5^ Department of Pediatrics, Tianjin Children’s Hospital, Tianjin, China

**Keywords:** miR-146b-3p, radioiodide therapy, sodium/iodide symporter, dedifferentiated thyroid cancer, *MUC20*

## Abstract

The dedifferentiation of differentiated thyroid cancer (DTC) is a challenging problem for radioactive iodine (^131^I) treatment, also known as radioiodine refractory differentiated thyroid cancer (RAIR-DTC). The purpose of this study was to further explore the mechanism of the redifferentiation of dedifferentiated thyroid cancer. Ineffective and effective groups of ^131^I therapy were analyzed and compared in both our clinical and TCGA samples. Whole-exome sequencing, mutation analysis, transcriptome analysis, and *in vitro* functional experiments were conducted. *FLG*, *FRG1*, *MUC6*, *MUC20*, and *PRUNE2* were overlapping mutation genes between our clinical cases, and the TCGA cases only appeared in the ineffective group. The expression of *miR-146b-3p* target *MUC20* was explored. The expression levels of *miR-146b-3p* and *MUC20* were significantly increased, and the inhibition of *miR-146b-3p* expression significantly inhibited proliferation and migration, promoted apoptosis, regulated the expression and location of thyroid differentiation-related genes, and sodium/iodide symporter (NIS) in dedifferentiated thyroid cancer cells (WRO). Thus, *miR-146b-3p* potentially targets *MUC20* participation in the formation of DTC dedifferentiation, resulting in resistance to ^131^I and the loss of the iodine uptake ability of DTC cancer foci, promoting refractory differentiated thyroid cancer. *miR-146b-3p* may be a potentially therapeutic target for the reapplication of ^131^I therapy in dedifferentiated thyroid cancer patients.

## Introduction

Thyroid cancer is the most common malignant tumor in the endocrine system. According to the most recent statistics, the incidence of thyroid cancer in the United States ranks sixth among female malignant tumors in 2019 (1). Differentiated thyroid cancer (DTC) is the most frequent subtype of thyroid cancer. Radioiodine (^131^I) is a classic radiotheragnostic agent used for the treatment of DTC following a thyroidectomy based on sodium-iodine symporter expression in normal and neoplastic thyroid tissue (2). Non-iodine uptake is an independent predictor of poor prognosis in DTC patients, and our previous study found that 30% of DTC patients with lung metastases did not take iodine (3). Surgery followed by radioactive iodine (^131^I) is effective in most DTC patients (4); however, some DTC dedifferentiate during treatment, and subsequently develop into radioiodine refractory differentiated thyroid cancer (RAIR-DTC). RAIR-DTC is a difficult problem associated with clinical treatment with rapid disease progression and poor prognosis.

Researches into relevant mechanisms are extremely important for the effective treatment of RAIR-DTC and some progress had been achieved. It has been reported that some DTC patients with metastases underwent degenerative changes in the morphology and function of tumor cells during ^131^I therapy. In addition, impaired expression of iodine key protein and a loss of iodine uptake eventually developed into dedifferentiated thyroid cancer (5). At this point, the DTC is RAIR-DTC, which cannot benefit from ^131^I therapy, and the survival time is much shorter than that of those with favorable iodine intake. For the RAIR-DTC therapy, restoring the ability of dedifferentiated thyroid cancer to uptake ^131^I may represent a potential solution. It is currently believed that the main mechanisms of ^131^I treatment of thyroid cancer are closely related to mutations in RAS, BRAFV600E, and TERT, activate the MAPK pathway, PI3KCA mutation activates the PI3K/Akt pathway, and β-catenin mutation activate the wnt/β-catenin pathway. They may cause cellular proliferation and dedifferentiation, which leads to an abnormal silence or decreased expression of key iodine uptake proteins (NIS, TSHR, TPO, and Tg) and thyroid transcription factors (e.g., TTF1 and TTF2) (6–9).

At present, several methods have been reported to improve the radioiodine uptake ability of dedifferentiated cancer cells, including prolonging the effective half-life of ^131^I, inducing cell redifferentiation, and transgene therapy (10). However, these methods are all associated with some shortcomings, including unstable iodine uptake, rapid iodine outflow, inadequate radiation efficiency, toxic side effects of molecular targeted drugs, and acquired drug resistance. Additionally, it is extremely important for radionuclide therapy of thyroid cancer to redifferentiate undifferentiated lesions or metastatic lesions and improve their uptake of ^131^I. Therefore, in order to identify a more effective strategy for the treatment of redifferentiation, it is necessary to further explore the mechanisms associated with the occurrence and development of RAIR-DTC.

## Materials and Methods

### Human Tissues and DNA Extraction

Six DTC patients accepted ^131^I therapy following total thyroidectomy which is a complete removal of the thyroid tumor and tissue, performed at Tianjin Medical University General Hospital from October 2016 to August 2018.

The iodine uptake capacity of tumor tissue was mainly evaluated using ^131^I whole-body scan (^131^I-WBS) after radioiodine therapy, A combination of ^131^I-WBS, serum Tg and TgAb levels, and imaging examinations were performed to assess RAIR-DTC.

**Figure 1:**
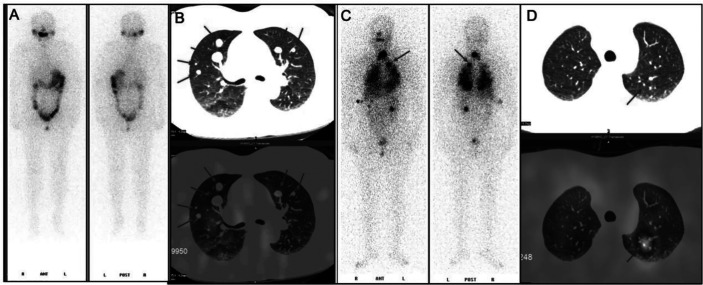
^131^I whole-body scan (^131^I-WBS) after radioiodine therapy. **(A, B)** ^131^I-WBS and CT image of a patient without iodine uptake of lung metastases after 131I remnant ablation successful. **(C, D)** ^131^I-WBS and CT image of a patient with iodine uptake of lung metastases.

Among these patients, three were RAIR-DTC patients without iodine uptake of lung metastases after 131I remnant ablation successful, all of which exhibited post-operative lung metastases (test group, **Figures 1A, B**). The other three cases possessed good iodine uptake capacity of lung metastases and were sensitive to radioiodine therapy (control group, **Figures 1C, D**). The primary surgical excision tissues were collected from these patients. All patients provided voluntary informed consent, and the study was approved by the ethics committee at our hospital. Genomic DNA was extracted and the concentration with a QIAamp^®^ DNA Micro kit (Qiagen, Heidelberg, Germany) in accordance with the manufacturer’s protocol. A Qubit system was used to quantify genomic DNA before library preparation.

### Cell Culture

According to our previous experiments and reported studies (5, 11, 12), the dedifferentiated thyroid cancer cell line WRO was selected for subsequent experiments. The WRO cell line was purchased from Sigma-Aldrich (Munich, Gemany) and the cells were cultured in DMEM medium (Gibco, USA) containing 10% fetal bovine serum (FBS, Gibco, USA), 1% penicillin (100 U/mL)/streptomycin (100 μg/mL; Gibco, USA), and were maintained in a 37°C incubator in a humidified, 5% CO_2_ atmosphere.

### TCGA Data Screening and Download of Mutation Information

A total of 491 thyroid cancer cases were selected from The Cancer Genome Atlas (TCGA) database based on the following screening criteria: 1) postoperative radioiodine therapy; 2) clear effect of iodine therapy; 3) complete information of gene mutation; and 4) complete information regarding microRNA (miRNA) expression data. Disease stabilization and progression were considered to be ineffective treatment (test group), and both a partial or complete responses were effective (control group). Thus, 167 ineffective and 324 effective patients were included. Subsequently, the single nucleotide variant (SNV) and insertion/deletion (Indel) mutations were downloaded and analyzed between the test and control groups. In addition, the mutation genes occurred only in the test group but not in control group were screened out, and recorded as a test-specific mutation gene set.

### Whole-Exome Sequencing and Mutation Analysis for Clinical Patients

After sheering and repairing the ends, the DNA samples were purified using AMPure XP beads (Beckman-Coulter, Indianapolis, USA). Subsequently, the paired-end adaptor was ligated, the samples were purified, the adaptor-ligated library was amplified, the amplified library was purified, and the quality and quantity were assessed using a Qubit Fluorometer (Invitrogen, Carlsbad, USA). A library hybridization kit, SeqCap EZ MedExome Enrichment kit (Roche NimbleGen, Madison, USA), was used to capture the target sequences. A whole-exon probe system was customized from Roche NimbleGen to capture target sequences, capture magnetic beads, and the elution of hybridization libraries. After amplifying the captured library by PCR, the constructed library was sequenced with an Illumina HiSeq Xten sequencer. The average sequencing depth of the tissue samples was 500X, which could detect mutations in tumor genes with very low frequency of 0.1%.

The sequencing data were filtered by SOAPnuke to remove the Adapter-containing sequences and low quality data. The quality of the raw data after filtering met the Q30 requirement of > 85%. BWA was used to compare the data to the human reference genome (hg19.fa). GATK was used to re-align the reads in the interval, calibrate and rearrange the alkali matrix quality values, and count the sequencing depth and coverage. An indel detection was conducted with GATK and VarScant, SNV was detected with MuTect and VarScan, and CNV was detected with CONTRA. The test-specific mutation gene set was also sifted out. Finally, they were annotated to a catalogue of somatic mutations in cancer (COSMIC, https://cancer.sanger.ac.uk/cosmic), the single nucleotide polymorphism database (dbSNP, https://www.ncbi.nlm.nih.gov/snp/), and the important genes related to the occurrence, development, treatment and prognosis of thyroid cancer, were selected out. A Venn diagram was drawn based on these genes and the TCGA data. Accordingly, the overlapping test-specific mutation genes were selected between TCGA cases and our clinical cases, and studied in in-depth.

### Identification of Participating Pathways Associated With Overlapping Mutation Genes

Using the Rectome database (https://www.reactome.org/), a free, open-source, curated and peer reviewed pathway database, we analyzed the pathways of the above overlapping genes.

### Transcriptome Analysis and Regulation Network Construction

The mRNA and miRNA expression data were obtained from TCGA data. After standardization with preprocessCore function package V3.5 (http://www.bioconductor.org/packages/release/bioc/html/preprocessCore.html), the differentially expressed genes (DEGs) and differentially expressed microRNAs (DEGs) were identified with the limma V3.18.13 software package (http://www.bioconductor.org/packages/2.13/bioc/html/limma.html) in the test group compared with control group. P < 0.05 and |log (fold-change)| > 2 were used as the threshold criteria. Using mirwall 3.0 (http://mirwalk.umm.uni-heidelberg.de/), we obtained the targeting relationship (gene-miRNA pairs) between the DEMs and overlapping genes. The protein-protein interactions (PPI) were analyzed *via* the STRING V10.0 database (http://string-db.org), and the PPI pairs of the overlapping mutation genes were screened out using more than 500 scores. Based on the above pairs, the regulation network was ultimately constructed and visualized using Cytoscape V3.5.1 software (http://www.cytoscape.org/download.php).

### *miR‐146b-3p* Inhibitors/nc Oligonucleotides and Cellular Transfection

The expression of *miR-146b-3p* and likely target *MUC20* were explored between our clinical cases and TCGA cases, and only appeared in the ineffective group. Then *miR‐146b-3p* inhibitors and individual nc oligonucleotide products (GenePharma, China) were synthesized. WRO cells were transfected with 100 nM of the indicated oligonucleotides separately using Lipo-fectamine 3000 (Invitrogen) according to the manufacturer’s protocol. At 24 to 48 h post-transfection, the resultant cells were used for functional assays. The remaining cells were harvested for quantitative polymerase chain reaction (qRT-PCR) analysis.

### RNA Extraction and RT-PCR Analysis

The expressions of the overlapping mutation genes *(FLG*, *FRG1*, *MUC6*, *MUC20*, and *PRUNE2)* were detected in the above clinical surgical excision tissues. The primer sequences of the genes are listed in **Table 1**. The total RNA was extracted using TRIzol (Invitrogen; Thermo Fisher Scientific, Inc., Waltham, USA). All of the specific primers were designed and synthesized by Takara Biotechnology Co., Ltd (Dalian, China), and are listed in **Table 1**. A PrimeScript^®^ 1st Stand cDNA Synthesis kit (Takara Biotechnology Co., Ltd., Dalian, China) and SYBR^®^ Premix Ex Taq™ kit (Takara Biotechnology Co., Ltd., Dalian, China) were used to conduct reverse transcription PCR (RT-PCR) in accordance with the manufacturer’s protocols. The reaction conditions for reverse transcription were as follows: 30°C for 10 min, 42°C for 60 min, and 95°C for 5 min. Finally, the relative expression value of the optical density was calculated for the target gene.

**Table 1:** Primer sequences of qRT-PCR of genes.

The name of the primer	Primer sequences (5′-3′)	
FLG	Forward	GCTGAAGGAACTTCTGGAAA
	Reverse	CATCAGAAGAAACTCAGTGAA
FRG1	Forward	GCTCCACACAAAGAAGTTGA
	Reverse	CTCTTGGTCCAATTGCATCT
MUC20	Forward	GATCACAACCTCAGCGAAGA
	Reverse	GAGCTGCTGCATCAGCCTTT
MUC6	Forward	CTCAACAAGGTGTGTGCAGA
	Reverse	GGAAAGGTCTCCTCGTAGTT
PRUNE2	Forward	CAGTTCAGTGCTCAGGGTTT
	Reverse	CCAAACTTGTCTGTAAATGCTT
ACTING	Forward	CTGGACTTCGAGCAAGAGAT
	Reverse	GATGTCCACGTCACACTTCA

### Cell Proliferation Assay

A WRO Cell Counting Kit-8 (CCK-8; Dojindo, Japan) assay was used to assess the level of cell proliferation/viability according to the manufacturer’s instructions. After an incubation with miR‐146b-3p inhibitors (100 nmol/μl) or the same volume of 0.1% DMSO (as control) for 24, 48, and 72 h, CCK8 reagent was added into each well, and the cells were incubated at 37°C for 2 h. Cell viability was measured by the level of absorbance (optical density) at a wavelength of 450 nm using a microplate reader.

### Cell Apoptosis Assay

WRO cells were seeded into six-well plates and treated with 100 nmol/μl *miR-146b-3p* inhibitors or the same volume of 0.1% DMSO (as control) for 72 h, then harvested and stained with Annexin V/propidium iodide (PI) staining kit (Beyotime, China) according to the manufacturer’s instructions. Finally, the apoptotic rates were obtained by flow cytometry.

### Colony Formation Assay

Different groups of WRO cells were cultured in six‐well plates. The cells were cultured in a 37°C incubator under humidified, 5% CO_2_ conditions for 7 to 10 days. The cells were then fixed and stained with a crystal violet solution for 30 min and images were obtained.

### Transwell Assay

Different groups of WRO cells were cultured, and a total of 1 × 10^5^ cells in 100 μl serum‐free DMEM were seeded into the upper chamber of the transwell plates precoated with Matrigel (Millipore). Complete DMEM medium was added to the lower chamber. After 24 h, cells that invaded the membrane were fixed, stained with a crystal violet solution for 2 min and counted.

### Wound‐Healing Assay

Different groups of WRO cells were transfected with miR‐146b-3p inhibitors or negative control and cultured until the cells reached 90% confluence. The wound was created using a 200 μl tip and the cells were continued to be cultured in DMEM medium for another 24 h or 48 h. Cellular migration was recorded by an inverted microscope and images were obtained.

### Immunofluorescence

WRO cells were transfected with miR‐146b-3p inhibitors or negative control and were fixed in 4% paraformaldehyde in phosphate-buffered saline. The cells were subsequently incubated with an anti-NIS antibody (Bioss, China) overnight at 4°C and subsequently incubated with a fluorescein-conjugated secondary antibody (CST, USA) for 2 h. The nuclei were treated with RNase- and stained with 20 mg/ml propidium iodide (PI; Beyotime, China). Fluorescence was observed using a fluorescence microscope (Nikon Instruments Inc., Melville, NY, USA).

### Western Blot

Different groups of WRO cells were seeded into 24-well plates with a cover glass over each well and cultured for 24 h. Total, membranous and cytoplasmic NIS and MUC20 proteins were extracted according to the standard steps of the protein extraction kits.

Lysates were quantified spectrophotometrically using the bicinchoninic-acid-based (BCA) method. Twenty-five micrograms of each sample was separated on gradient polyacrylamide gels and transferred onto polyvinyldifluoride membranes. Membranes were incubated with a primary antibody (anti-NIS, Bioss, China; MUC 20, Abnova, USA; MET, abcam, UK; MET pY1234/5, CST, USA; β‐actin, CST, USA) overnight at 4°C in TTBS/milk. The samples were subsequently incubated with horseradish peroxidase-conjugated secondary anti-mouse antibodies (CST, USA). The protein was visualized using an enhanced chemiluminescence Western blot detection system (Thermo Fisher Scientific). Multiplication of the intensity and area of protein bands indicated the relative levels of protein expression.

### Radioiodide Uptake Assay (RAIU)

WRO cells were transfected with miR‐146b-3p inhibitors or negative control and were seeded into 24-well plates, the cells were washed with ice-cold modified Hanks’ balanced salt solution (HBSS) three times and then incubated at 37°C with 500 μl buffered HBSS containing 2 μCi Na^125^I for 30 min. Then, radioactive medium was aspirated and cells were washed (×3) with 1ml of ice-cold HBSS for 1min. Cells were harvested using trypsin and counted with a hemacytometer. The radioactivity was measured with a gamma counter. The radioactivity was normalized to the number of cells present at the time of the assay as cpm every 10^6^ cells.

### Statistical Analysis

SPSS V23.0 software (SPSS Inc., Chicago, USA) was used for all statistical analyses, and the data were expressed as the mean ± SD. A t-test was used to compare the differences between two groups, and P < 0.05 was considered to be statistically significant.

## Results

### The Expression of miR-146b-3p Target MUC20 Were Explored by Bioinformatics Analysis

**Figure 2:**
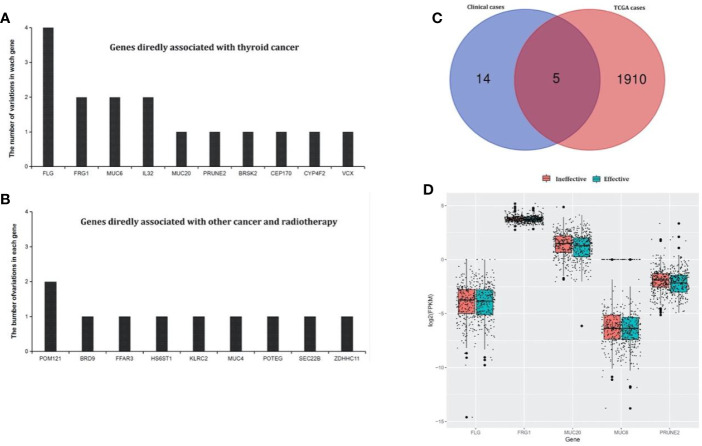
Test-specific mutation genes directly and indirectly associated with thyroid cancer identified in our clinical samples. **(A)** Genes in the annotated database that showed clear evidence of being associated with thyroid cancer. There were 10 genes that were directly related to thyroid cancer. **(B)** Genes in the annotated database that showed clear evidence of being associated with other cancers and radiotherapy. There were nine genes that may be related to thyroid cancer (associated with other cancers and radiation therapy). **(C)** The Venn diagram of test-specific mutation genes between our clinical and TCGA cases. There were five overlapping test-specific mutation genes in TCGA and our clinical cases (*FLG*, *FRG1*, *MUC6*, *MUC20*, and *PRUNE2*). **(D)** Differential expression analysis of the overlapping genes in the TCGA cases between the test an control groups. *MUC20* and *FRG1* were differentially expressed.

From the TCGA cases, a total of 7,335 mutation genes were identified in 491 thyroid cancer samples. It was further found that there were 1,915 specific mutation genes in test group compared with the control group. In our clinical cases, there were 55 test-specific variation sites in the test that occurred in 41 genes. After annotating to COSMIC and dbSNP, 10 genes were directly related to thyroid cancer, and 9 genes may have been related to thyroid cancer (associated with other cancers and radiation therapy). The 19 genes were listed in **Figures 2A, B**, as well as a Venn diagram and the above 1915 test-specific mutation genes are shown in **Figure 2C**. Five test-specific mutation genes were overlapped in TCGA and our clinical cases (i.e., *FLG*, *FRG1*, *MUC6*, *MUC20*, and *PRUNE2*).

**Figure 3:**
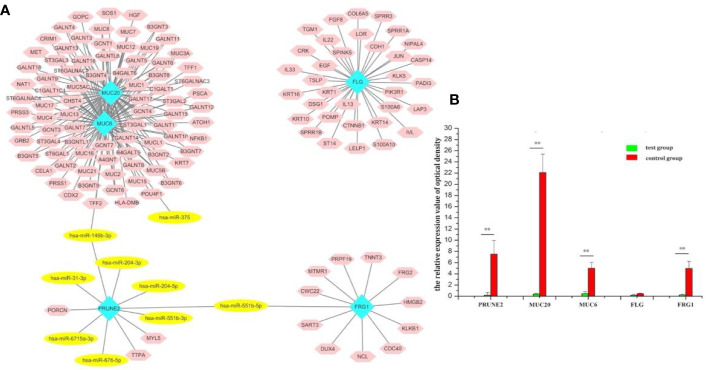
Screening of the target genes and exploration of the miR-146b-3p target MUC20. **(A)** The regulated network of the overlapping mutation genes. The network contained four functional groups, recorded as: *MUC6*-*MUC20* group, *PRUNE2* group, *FLG* group, and *FRG1* group. Interestingly, *miR-146b-3p* was connected to the *MUC6*-*MUC20* group and *PRUNE2* group, and *miR-551b-5p* linked the *PRUNE2* and *FRG1* groups together. **(B)** RT-PCR results regarding the expression of the overlapping mutation genes. *MUC20* was significantly highly expressed. (**p < 0.01).

The gene-miRNA regulation network was further analyzed. Among the TCGA cases, the results of the differential expression analysis of the above overlapping mutation genes were compared between the test group and control group, and *MUC20* and *FRG1* were found to be differentially expressed (**Figure 2D**). Simultaneously, 10 DEMs were identified in the test group compared with control group, including *miR-204*, *miR-7-2*, and *miR-551b* (**Table 2**). Furthermore, the gene-miRNA pairs were obtained by miRwall 3.0 and exhibited in **Table 3** (e.g., *miR-146b-3p*-*MUC20* and *hsa-miR-146b-3p*-*PRUNE2*). After screening with STRING V10.0, 196 PPI pairs were obtained. Eventually, the regulated network was constructed and presented in **Figure 3A**. The network contained four functional groups, which were recorded as the: 1) *MUC6*-*MUC20* group; 2) *PRUNE2* group; 3) *FLG* group; and 4) *FRG1* group. Interestingly, *hsa-miR-146b-3p* connected the *MUC6*-*MUC20* group and *PRUNE2* group. Similarly, *hsa-miR-551b-5p* linked the *PRUNE2* group and *FRG1* group together.

**Table 2:** The DEMs identified in test group compared with control group among TCGA cases.

MiRNA	LogFC	AveExpr	T Value	P Value
hsa-mir-204	0.863062063	6.400199623	3.88062297	0.000120147
hsa-mir-7-2	0.862249179	3.948694306	3.800144007	0.000164985
hsa-mir-551b	-0.831391274	6.423744887	-3.54704631	0.000431436
hsa-mir-7-3	0.718090127	2.127805746	3.40904965	0.000711975
hsa-mir-676	0.527504431	3.443443543	3.378238087	0.000794446
hsa-mir-6715a	0.803311975	2.771116168	3.299026227	0.001049064
hsa-mir-146b	-0.780099202	16.03598508	-3.161258887	0.001679494
hsa-mir-31	-0.746292503	7.960612802	-3.151052959	0.00173794
hsa-mir-1179	0.65710791	3.225364061	3.096506992	0.002083391
hsa-mir-375	-0.662702182	14.03006948	-2.844438491	0.004656558

DEMs, differential expression microRNAs; LogFC, Log (fold-change); AveExpr, average expression.

**Table 3:** The gene-miRNA pairs between DEMs and overlapped mutation genes.

MiRNA	Gene	LogFC_miRNA	LogFC_gene	Position
hsa-miR-146b-3p	MUC20	-0.780099202	-0.519617606	CDS
hsa-miR-146b-3p	PRUNE2	-0.780099202		CDS
hsa-miR-204-3p	PRUNE2	0.863062063		CDS
hsa-miR-204-5p	PRUNE2	0.863062063		CDS
hsa-miR-31-3p	PRUNE2	-0.746292503		CDS
hsa-miR-375	MUC6	-0.662702182		CDS
hsa-miR-551b-3p	PRUNE2	-0.831391274		3’UTR
hsa-miR-551b-5p	PRUNE2	-0.831391274		3’UTR
hsa-miR-551b-5p	FRG1	-0.831391274	-0.70594252	CDS
hsa-miR-6715a-3p	PRUNE2	0.803311975		CDS
hsa-miR-676-5p	PRUNE2	0.527504431		3’UTR

DEMs, differential expression microRNAs; LogFC, Log (fold-change); LogFC_miRNA, the logFC of miRNA; LogFC_gene, the logFC of gene; CDS, coding sequence; 3’UTR, 3’ untranslated region.

**Figure 4:**
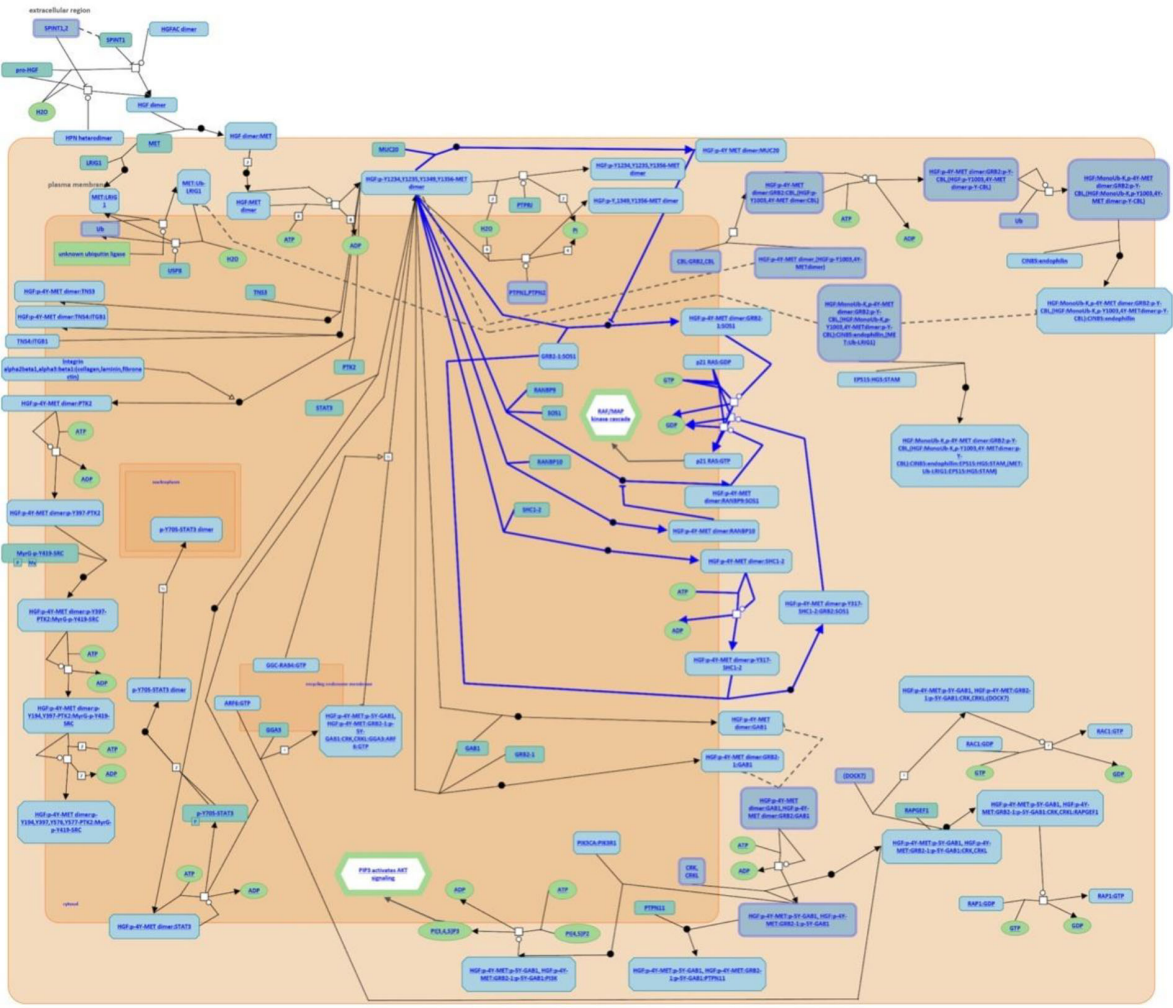
Participating pathways analysis. Only MUC20 was found to participate in the pathways, “negative regulation of MET activity” and “MET activates RAS signaling”.

After analysis of the above overlapping mutation genes, only *MUC20* was found to be participating in the pathways, “negative regulation of MET activity” and “MET activates RAS signaling” (**Figure 4**). *FLG*, *FRG1*, *MUC6*, and *PRUNE2* were not found to participate in any pathways.

Furthermore, the expression overlapping mutation genes in thyroid cancer was validated by qRT-PCR. After verification by RT-PCR, *FRG1*, *MUC6*, *MUC20*, and *PRUNE2* were significantly different between the FTC-133 and WRO groups **(** **Figure 3B**). In particular, *MUC20* was the most remarkable (p < 0.01). Through the above bioinformatics analysis, the expression of miR-146b-3p target MUC20 were explored.

### The Overexpression of *miR-146b-3p* in Dedifferentiated Thyroid Cancer Cells

**Figure 5:**
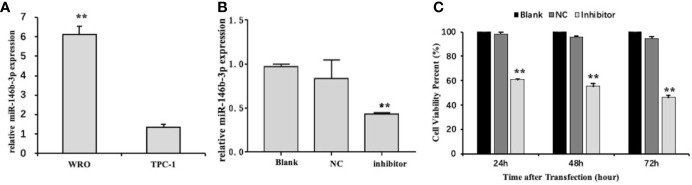
The level of miR-146b-3p overexpression in dedifferentiated thyroid cancer cells. **(A)** Expression of miR-146b-3p in thyroid cancer cells with different degrees of differentiation. The level of miR‐146b-3p expression was significantly increased in the dedifferentiated thyroid cancer WRO cell lines. **(B)** Expression of miR-146b-3p following transfection with an inhibitor. The miR‐146b-3p expression was substantially lower than that of the control groups following transfection with inhibitors (**p < 0.01). **(C)** Effects of anti-miR‐146b-3p on thyroid cancer cell proliferation was determined by CCK‐8 cell proliferation assays. Data were represented as the means ± SD.

To investigate the possible function of *miR‐146b-3p* in the regulation of dedifferentiated thyroid cancer, we first examined the level of *miR‐146b-3p* mRNA expression in differentiated and dedifferentiated thyroid cancer cell lines (TPC-1 and WRO) by qRT-PCR. Interestingly, *miR‐146b-3p* expression was significantly increased in dedifferentiated thyroid cancer WRO cell lines (**Figure 5A**). Presumably, it is possible to associate *miR‐146b-3p* expression with the dedifferentiation of differentiated thyroid cancer.

To confirm our hypothesis, we established *miR‐146b-3p* knockdown models. As a result, the transfected cells carrying *miR‐146b-3p* inhibitors (*anti-miR‐146b-3p*)/nc are considered ideal tools for *miR‐146b-3p* research.

**Figure 5B** shows that *miR‐146b-3p* expression is apparently lower than the nc control upon the transfection of inhibitors. This finding indicates that *miR‐146b-3p* inhibitors have a remarkable effect on a *miR‐146b-3p* knockdown.

### *Anti-miR‐146b-3p* Inhibited Cell Proliferation, Invasion, and Migration, as Well as Induced Apoptosis in Dedifferentiated Thyroid Cancer Cells

To analyze the role of *miR‐146b-3p* in regulating the proliferation of WRO cells, a CCK-8 assay was used. As indicated in **Figure 5C** *miR‐146b-3p* inhibitors significantly suppressed the proliferation of WRO cells in a time-dependent manner. Following transfection with *miR‐146b-3p* inhibitors for 24 h, 48 h, and 72 h, the viability rates of WRO cells were 60.79% ± 0.34%, 55.44% ± 2.17%, and 46.38% ± 1.64%, respectively (*P* < 0.01). These findings revealed that *miR‐146b-3p* overexpression could significantly increase the proliferation of dedifferentiated thyroid cancer cells.

**Figure 6:**
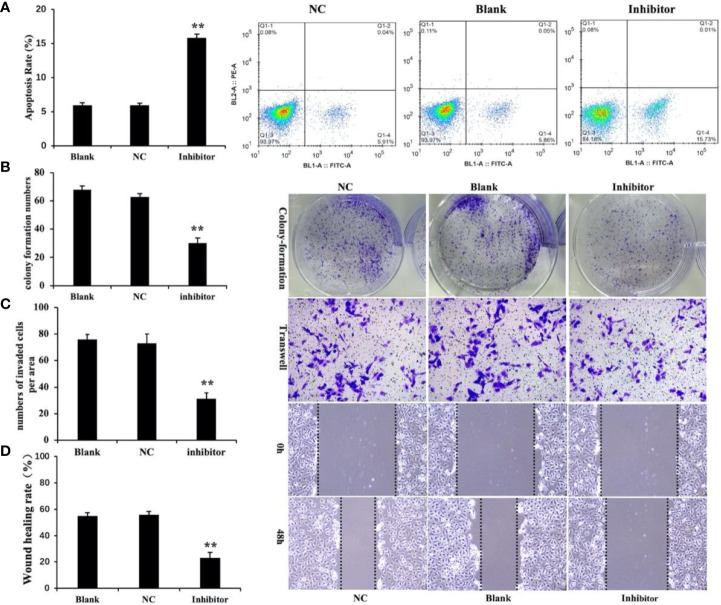
Anti-miR‐146b-3p inhibited dedifferentiated thyroid cancer cell proliferation, invasion, migration, and induction of apoptosis. **(A)** The effects of anti-miR‐146b-3p on thyroid cancer cell apoptosis was measured by flow cytometry. Data were represented as the means ± SD. **(B–D)** The effects of anti-miR‐146b-3p on thyroid cancer cell proliferation, invasion, and migration were determined by colony formation, transwell, and wound healing assays. The results showed that treatment with miR‐146b-3p inhibitors significantly suppressed the proliferation and invasion of WRO cells (**p < 0.01).

Cell apoptosis was measured by flow cytometry. The results showed that the level of apoptosis was significantly higher in the *miR‐146b-3p* inhibitors transfection group compared to that of the control groups (**Figure 6A**). In the three groups of WRO cells, apoptosis rates of 15.73% ± 1.2%, 5.89% ± 0.35%, 5.86% ± 1.4%, and were observed in the *miR‐146b-3p* inhibitors transfection group and control groups, respectively (P < 0.01). The results revealed that *anti-miR‐146b-3p* could significantly increase apoptosis in dedifferentiated thyroid cancer cells.

We studied the effects of *miR‐146b-3p* on cell growth and colony formation by a colony‐formation assay. The results revealed that the number of *miR‐146b-3p* inhibitor‐transfected cells was significantly lower than that of the control groups (**Figure 6B**). This indicated that *miR‐146b-3p* overexpression could significantly increase growth in dedifferentiated thyroid cancer cells.

Cell migration and invasion are critical for tumor growth. To explore the effect of *miR‐146b-3p* on WRO cell migration and invasion, we performed both wound‐healing and transwell assays. The results showed that inhibition miR‐146b-3p expression obviously suppressed the migratory and invasive ability of WRO cells compared to that of the control groups. This revealed that *miR‐146b-3p* overexpression could significantly promote WRO cell migration and invasion (**Figures 6C, D**).

Together, these findings suggest that the upregulation of miR‐146b-3p may contribute to dedifferentiated thyroid cancer progression by promoting cell proliferation and metastasis.

### *Anti-miR‐146b-3p* Upregulated NIS Expression in Dedifferentiated Thyroid Cancer Cells

**Figure 7:**
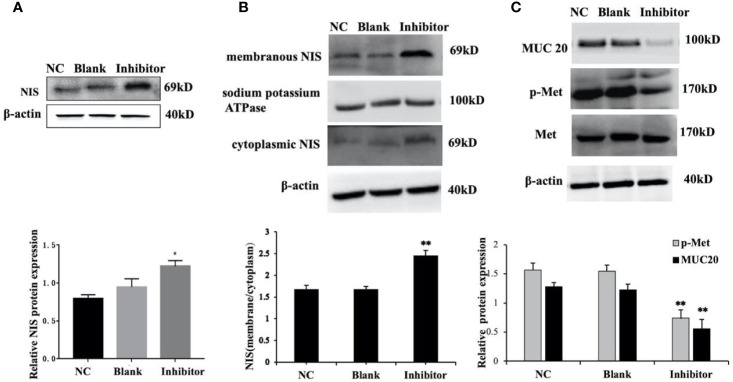
*miR-146b-3*p*/MUC20/MET* signaling pathway modulates the sodium/iodide symporter (NIS)-mediated radioiodide uptake in dedifferentiated thyroid cancer cells. **(A, B)** The expressions of NIS protein in total, cytomembrane and cytoplasm in WRO cells with *Anti-miR‐146b-3*p in time course experiments were determined by western blot analysis with quantification. The results showed that the level of NIS protein expression in the miR‐146b-3p inhibitor‐transfected cells were notably increased compared to that of the control groups (*p < 0.05; **p < 0.01). **(C)** The expression of MUC20, MET, p-MET were inhibited by *Anti-miR‐146b-3*p and was related to MET Signaling pathways.

**Figure 8:**
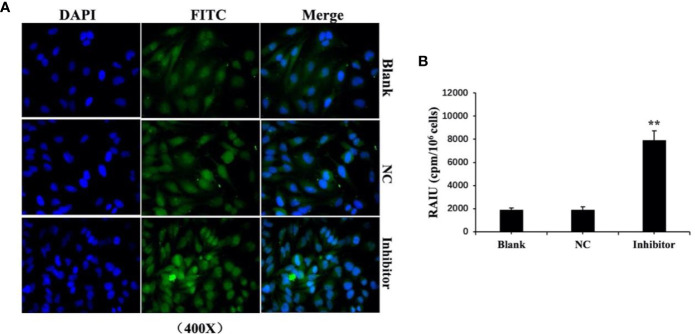
Anti-miR‐146b-3p upregulated the expression and translocation of sodium/iodide symporter (NIS)-mediated radioiodide uptake in dedifferentiated thyroid cancer cells. **(A)** Effect of anti-miR‐146b-3p on NIS protein translocation by immunofluorescence. The results showed that anti-miR‐146b-3p increased both cytoplasmic and cytomembrane-located NIS expression, the latter of which being more significant. **(B)** Radioactive iodine (RAIU) uptake in WRO cells was determined. The results showed that anti-miR‐146b-3p increased the radioactive iodine uptake (**p < 0.01).

We previously found that *anti-miR‐146b-3p* caused significant inhibition of WRO cell proliferation, growth, migration, as well as the induction of apoptosis. We subsequently detected the level of NIS protein expression by Western blot. The results showed that the level of NIS protein (total and membranous/cytoplasmic) expression in *miR‐146b-3p* inhibitor‐transfected cells were obviously increased compared to that of the control groups (**Figures 7A, B**). We further investigated the effect of *anti-miR‐146b-3p* on NIS protein translocation by immunofluorescence. The results showed that *anti-miR‐146b-3p* increased both cytoplasmic and cytomembrane-located NIS expression, the latter of which was more significant (**Figure 8A**).

### *miR-146b-3*p*/MUC20/MET* Signaling Pathway Modulates the NIS-Mediated Radioiodide Uptake in Dedifferentiated Thyroid Cancer Cells

We further explore and study the mechanism of *miR-146b-3p* involving in dedifferentiation of thyroid cancer. The results suggested that MUC*20*, MET, and phosphorylation of MET (p-MET) significantly inhibited after inhibiting the *miR-146b-3p* (**Figure 7C**). Moreover, we found that inhibition of *miR-146b-3p* expression improve NIS-mediated radioiodide uptake ability compared to the control groups (**Figure 8B**).

The above results indicated that *miR-146b-3p*/*MUC20/MET* signal pathways may play vital roles in NIS expression and radioiodide uptake by inhibition of *mi*R-146b-3p expression to induce redifferentiation of dedifferentiated thyroid cancer cells.

## Discussion

Radioiodine refractoriness is primarily related to the NIS expression of the thyroid cancer cells. For RAIR-DTC, it is mainly due to a decrease in the level of NIS expression or the appearance of heterotopic expression outside the basement membrane of the thyroid cancer cells. This makes the treatment of ^131^I ineffective, which causes disease progression. The NIS is a membrane glycoprotein that can transport two sodium ions and one iodide ion into the cytosol *via* extracellular fluid (8, 13). Since radioiodine also can be taken up by the NIS, radioiodine can be used to visualize or selectively kill DTC cells. To upregulate the expression of NIS, restoration or improvement of the treatment of ^131^I is an important measure for clinical RAIR-DTC patients.

Recently, an in‐depth study of miRNAs showed that miRNAs can function as tumor suppressor genes or oncogenes, which are closely related to the formation and development of thyroid cancer and has become a topic of research interest in the field of cancer (14–16). Some miRNAs have been found to participate in the regulation of tumor radiosensitivity by regulating the expression of target genes and vital signaling pathways (17, 18). In our study, we sequenced and analyzed the pathological specimens of RAIR-DTC patients, pathogenic *miR-146b-3p* was selected as the key research objective. And through the bioinformatics analysis, the expression of miR-146b-3p target MUC20 were explored. Whether it is involved in dedifferentiation of DTC is largely unknown.

Recent studies have shown that miR-146b is related to the occurrence, development, proliferation, invasion, and metastasis of thyroid cancer (19–21). Several reports have confirmed *miR-146b* to be highly expressed in patients with a high risk of BRAF V600E mutations and advanced PTC (P = 0.001), suggesting that miR-146b is a marker of recurrence or metastasis in PTC patients (20). Riesco-Eizaguirre et al (22). reported that miR-146b-3p could directly bind to the 3’-UTR of the iodine metabolism-related proteins, PAX8, and NIS, resulting in decreased NIS expression and iodine uptake. In our study, we first confirmed that *miR-146b-3p* was highly expressed in WRO cells and positively correlated with the degree of DTC malignancy, which could lead to decrease NIS protein expression. We further investigated the biological function of *miR-146b-3p* in a WRO model for tumor progression and metastasis. Combined with the results of MTS, flow cytometry, colony-forming, wound‐healing and transwell assays, we inferred that *anti-miR‐146b-3p* had a significant inhibitory effect on tumor growth, invasion, migration, and induction of apoptosis in dedifferentiated thyroid cancer cells. Furthermore, the western blot results combined with immunofluorescence and Radioactive iodine uptake, inferred that *anti-miR-146b-3p* could increase NIS-induced iodide uptake by upregulating the expression and trafficking of NIS to the cell surface of WRO cells. Similar results were observed in previous studies (19, 22, 23). Therefore, *miR-146b-3p* may play an important role in the process of DTC dedifferentiation and represent a potential target for the treatment of RAIR-DTC.

MUC is primarily encoded by its polypeptide gene (*MUC* gene), a high molecular weight glycoprotein produced by epithelial cells, which lubricates and protects the mucosa (24). At present, more than 20 kinds of MUC have been reported, mainly including the MUC1-20 subtype. Recent studies have shown that different subtypes of MUC play an important role in the occurrence and development of various cancers (25). MUC20 is over-expressed in many cancers, and has been shown to regulate cell growth, differentiation, metastasis, adhesion, and invasive immune surveillance. MUC20 is also an independent prognostic factor for the poor survival rate of malignant tumors (26, 27). In thyroid cancer, studies have found that MUC1, MUC4, and MU15 are overexpressed in PTC. MUC1 overexpression has been studied as a key molecular event in the pathogenesis of invasive PTC. For PTC patients with a BRAF V600E mutation, MUC1 is an important oncogene that could be used as a prognostic indicator (28). The study by Nam et al. found that elderly, multifocal PTC patients with distant metastasis were often accompanied by the overexpression of MUC15 (29). MUC15 could be used as a prognostic marker and represent a potential new therapeutic target for PTC and marker for evaluating prognosis. At present, there have been no reports regarding MUC20 in dedifferentiated thyroid cancer. Our findings reveal that *MUC20* is prone to mutation in the group for which radioiodine therapy was ineffective. Moreover, *MUC20* exhibited significantly high expression in dedifferentiated thyroid cancer cells. Therefore, MUC20 may be related to the formation of DTC dedifferentiation.

To further explore and study the mechanism of resistance to treatment with ^131^I and DTC dedifferentiation, we performed a pathway analysis and found that miR-146b-3p*/MUC20* participated in the pathways, “negative regulation of MET activity”, and “MET activates RAS signaling”. The MET pathway is insensitive to targeted drugs (e.g., BRAF or MEK inhibitors) in refractory thyroid cancer, which can cause a BRAFV600E mutation by activating the downstream PI3K/Akt pathway (30, 31). Other studies have shown that combined treatment with MET and MAPK pathway inhibitors can effectively inhibit the proliferation and differentiation of thyroid cancer cells (32). Our results suggested that MUC*20*, MET, and phosphorylation of MET (p-MET) significantly inhibited after inhibiting the *miR-146b-3p* (**Figure 7C**). Moreover, inhibition of *miR-146b-3p* expression could improve NIS-mediated radioiodide uptake ability compared to the control groups (**Figure 8B**). Therefore, it indicated that *miR-146b-3p*/*MUC20/MET* pathways may play important role in NIS expression and radioiodide uptake by inhibition of *mi*R-146b-3p expression to induce redifferentiation of dedifferentiated thyroid cancer cells. These studies indicate that the *miR-146b-3p/MUC20/MET pat*hway may be related to the occurrence and development of dedifferentiation of thyroid cancer. Thus, inhibiting MET pathway may become a new strategy for the treatment of dedifferentiation of thyroid cancer. Consequently, we suspect that the *miR-146b-3p*/*MUC20*/MET signaling pathway the targeting relationship of might play a role in the redifferentiation of iodine-refractory thyroid cancer.

In our study, we assessed the therapeutic potential of *miR-146b-3p* small-molecule inhibitor (*anti-miR-146b-3p*) for RAIR-DTC using WRO cell lines. WRO is the same as WRO82-1, and some websites stated that WRO and WRO82-1 are the same line (https://www.wikidata.org/wiki/Q54994417, https://web.expasy.org/cellosaurus/CVCL_0582). therefore, WRO cell line were selected based on Shang and Ashley N Reeb studies (11, 12), which showed that the expression of NIS and the radioiodine uptake capacity of thyroid cancer cells in WRO were reduced.

Overall, our study synthesized relevant findings, and demonstrated that *miR-146b-3p* was related to the formation of DTC dedifferentiation, resulting in decreased NIS expression and iodine uptake. Combined with a bioinformatics analysis, we put forth the following reasonable hypothesis: the regulation of the MET pathway by *miR-146b-3p* likely targets *MUC20*, which participates in the formation of DTC dedifferentiation. This results in resistance to ^131^I and loss of the ability for DTC cancer foci to uptake iodine, becoming refractory differentiated thyroid cancer.

The following factors make the results of this study significant: 1) due to the difficulties associated with AIR-DTC, we analyzed and screened the pathological tissue samples of RAIR-DTC patients, verified the biological function of the target gene *in vitro*, and predicted related pathways that might represent potential biomarkers of refractory thyroid cancer; 2) the *miR-146b-3p*/*MUC20*/MET signaling pathway provided a novel targets for the clinical treatment of RAIR-DTC, and the possibility of its further application in radioactive therapy of dedifferentiated thyroid-derived tumors was explored; 3) we demonstrated the possibility that *miR-146b-3p* and *MUC20* were likely oncogenes of refractory thyroid cancer, two novel biological markers for screening refractory thyroid cancer, and potential drug targets for gene therapy. Therefore, these results have important clinical significance and provide possible avenues for clinical transformation.

However, there are also some limitations associated with the present study, including the small clinical sample size. Clinical samples were difficult to obtain as a result of the long interval between the determination of clinical effectiveness of radioiodine therapy and surgical excision. The associated timing can take at least six months or even longer than one year, and there is a relatively small number of ineffective patients. We collected samples throughout the past three years, excluding those with severe nucleic acid degradation, unclear therapeutic effects, lost to follow-up, and exhibiting non-lung metastasis. Only three suitable samples were screened out in the experimental group. To further clarify and verify the associated mechanism, the targeting relationship of *miR-146b-3p*/*MUC20*/MET signaling pathway should be further validated. In addition, the role and mechanism of the targets in this pathway should be studied in-depth in the context of redifferentiation of iodine-refractory thyroid cancer.

## Conclusion

In conclusion, our present findings show that *anti-miR-146b-3p* could restore radioiodine uptake in dedifferentiated thyroid cancer by up-regulating NIS expression and translocation to the cell membrane *in vitro* by targeting MUC20 through MET Signaling pathways. This provides valuable evidence that *anti-miR-146b-3p* may represent a promising anticancer drug for the treatment of dedifferentiated thyroid cancers.

## Data Availability Statement

The original contributions presented in the study are publicly available. This data can be found here: https://www.ncbi.nlm.nih.gov/Traces/study/?acc=PRJNA683915.

## Ethic Statement

The studies involving human participants were reviewed and approved by The Ethical Committee of Tianjin Medical University General Hospital. The patients/participants provided their written informed consent to participate in this study.

## Author Contributions

JT, CC, and SH designed the project. SH, XX, and JZ consulted literature, collected human tissues, and performed the cell experiments. SH wrote the main manuscripts.SH and CW analyzed data. NL conducted bioinformatics analysis. ZM supervised experiments. All authors contributed to the article and approved the submitted version.

## Funding

This investigation was supported by the Program of Tianjin Science and Technology Plan 18ZXDBSY00170 (awarded to CC).

## Conflict of Interest

The authors declare that the research was conducted in the absence of any commercial or financial relationships that could be construed as a potential conflict of interest.

## References

[1] Siegel R Miller K Jemal A Cancer Statistics. 2019. CA Cancer J Clin (2019) 69(1):7–34. 10.3322/caac.21551 30620402

[2] Clerc J Verburg FA Avram AM Giovanella L Hindié E Taïeb D Radioiodine treatment after surgery for differentiated thyroid cancer: a reasonable option. Eur J Nuclear Med Mol Imaging (2017) 44(6):918–25. 10.1007/s00259-017-3654-z 28213773

[3] Wang R Zhang Y Tan J Zhang G Zhang R Zheng W Analysis of radioiodine therapy and prognostic factors of differentiated thyroid cancer patients with pulmonary metastasis: An 8-year retrospective study. Medicine (2017) 96(19):e6809. 10.1097/MD.0000000000006809 28489758 PMC5428592

[4] Cabanillas ME McFadden DG Durante C Thyroid cancer. Lancet (2016) 388(10061):2783–95. 10.1016/S0140-6736(16)30172-6 27240885

[5] Feng F Wang H Hou S Fu H Re-induction of cell differentiation and (131)I uptake in dedifferentiated FTC-133 cell line by TSHR gene transfection. Nuclear Med Biol (2012) 39(8):1261–5. 10.1016/j.nucmedbio.2012.07.004 22898315

[6] Mendes CS De CDP Vaisman M New perspectives on the treatment of differentiated thyroid cancer. Arquivos Brasileiros Endocrinol Metabol (2007) 51(4):612–24. 10.1590/S0004-27302007000400017 17684624

[7] Cheng L Jin Y Liu M Ruan M Chen L HER inhibitor promotes BRAF/MEK inhibitor-induced redifferentiation in papillary thyroid cancer harboring BRAFV600E. Oncotarget (2017) 8(12):19843–54. 10.18632/oncotarget.15773 PMC5386727 28423638

[8] Moon HC Byeong-Cheol A Redifferentiation of Radioiodine Refractory Differentiated Thyroid Cancer for Reapplication of I-131 Therapy. Front Endocrinol (2017) 8:260. 10.3389/fendo.2017.00260 PMC5649198 29085335

[9] Larson SM Osborne JR Grewal RK Tuttle RM Redifferentiating Thyroid Cancer: Selumetinib-enhanced Radioiodine Uptake in Thyroid Cancer. Mol Imaging Radionuclide Ther (2017) 26(Suppl 1):80–6. 10.4274/2017.26.suppl.09 PMC5283711 28117292

[10] Tesselaar MH Smit JW Nagarajah J Netea-Maier RT Plantinga TS Pathological processes and therapeutic advances in radioiodide refractory thyroid cancer. J Mol Endocrinol (2017) 59(4):R141–54. 10.1530/JME-17-0134 28931558

[11] Shang HX Zhao JY Yao JM Wang HJ Domg JJ Liao L Nevirapine Increases Sodium/Iodide Symporter-Mediated Radioiodide Uptake by Activation of TSHR/cAMP/CREB/PAX8 Signaling Pathway in Dedifferentiated Thyroid Cancer. Front Oncol (2020) 10:404. 10.3389/fonc.2020.00404 32300552 PMC7145398

[12] Reeb AN Ziegler A Lin R-Y Characterization of human follicular thyroid cancer cell lines in preclinical mouse models. Endocrine Connections (2016) 5:41–54. 10.1530/EC-15-0114 26830329 PMC5002955

[13] Ahn BC Sodium iodide symporter for nuclear molecular imaging and gene therapy: from bedside to bench and back. Theranostics (2012) 2(4):392–402. 10.7150/thno.3722 22539935 PMC3337731

[14] Shen CT Qiu ZL Song HJ Wei WJ Luo QY miRNA-106a directly targeting RARB associates with the expression of Na(+)/I(-) symporter in thyroid cancer by regulating MAPK signaling pathway. J Exp Clin Cancer Res (2016) 35(1):101. 10.1186/s13046-016-0377-0 27342319 PMC4919890

[15] Ortiz IMDP Barros-Filho MC Dos Reis MB Beltrami CM Marchi FA Kuasne H Loss of DNA methylation is related to increased expression of miR-21 and miR-146b in papillary thyroid carcinoma. Clin Epigenet (2018) 10(1):144. 10.1186/s13148-018-0579-8 PMC6245861 30454026

[16] Chou CK Liu RT Kang HY MicroRNA-146b: A Novel Biomarker and Therapeutic Target for Human Papillary Thyroid Cancer. Int J Mol Sci (2017) 18:636. 10.3390/ijms18030636 PMC5372649 28294980

[17] Nikiforova MN Gandhi M Gandi M Kelly L Nikiforov YE MicroRNA dysregulation in human thyroid cells following exposure to ionizing radiation. Thyroid (2011) 21(3):261–6. 10.1089/thy.2010.0376 PMC3048775 21323591

[18] Penha RCC Pellecchia S Pacelli R Pinto LFR Fusco A Ionizing Radiation Deregulates the MicroRNA Expression Profile in Differentiated Thyroid Cells. Thyroid (2018) 28(3):407–21. 10.1089/thy.2017.0458 29397781

[19] Yu C Zhang L Luo D Yan F Liu J Shao S MicroRNA-146b-3p promotes cell metastasis by directly targeting NF2 in human papillary thyroid cancer. Thyroid (2018) 28(12):1627–41. 10.1089/thy.2017.0626 PMC6308293 30244634

[20] Chou C Chen R Chou F Chang H Chen Y Lee Y miR-146b is Highly Expressed in Adult Papillary Thyroid Carcinomas with High Risk Features Including Extrathyroidal Invasion and the BRAF V600E Mutation. Thyroid (2010) 20(5):489–94. 10.1089/thy.2009.0027 20406109

[21] Pan Y Yun W Shi B Cui R Liu C Ding Z Downregulation of miR-146b-5p via iodine involvement repressed papillary thyroid carcinoma cell proliferation. J Mol Endocrinol (2020) 65(2):1–10. 10.1530/JME-19-0198 32302969

[22] Riesco-Eizaguirre G Wert-Lamas L Perales-Patón J Sastre-Perona A Fernández LP Santisteban P The miR-146b-3p/PAX8/NIS regulatory circuit modulates the differentiation phenotype and function of thyroid cells during carcinogenesis. Cancer Res (2015) 75(19):4119–30. 10.1158/0008-5472.CAN-14-3547 26282166

[23] Wächter S Wunderlich A Greene BH Roth S Elxnat M Fellinger SA Selumetinib Activity in Thyroid Cancer Cells: Modulation of Sodium Iodide Symporter and Associated miRNAs. Int J Mol Sci (2018) 19(7):pii: E2077. 10.3390/ijms19072077 PMC6073679 30018229

[24] Corfield AP Mucins: a biologically relevant glycan barrier in mucosal protection. Biochim Biophys Acta (BBA)-General Subj (2015) 1850(1):236–52. 10.1016/j.bbagen.2014.05.003 24821013

[25] Bhatia R Gautam S Cannon A Thompson C Hall B Aithal A Cancer-associated mucins: role in immune modulation and metastasis. Cancer Metastasis Rev (2019) 38(1–2):223–36. 10.1007/s10555-018-09775-0 PMC6614013 30618016

[26] Chen ST Kuo TC Liao YY Lin MC Tien YW Huang MC Silencing of MUC20 suppresses the malignant character of pancreatic ductal adenocarcinoma cells through inhibition of the HGF/MET pathway. Oncogene (2018) 37(46):6041–53. 10.1038/s41388-018-0403-0 PMC6237765 29993037

[27] Chen C Wang S Chen C Huang M Hung J Huang H MUC20 overexpression predicts poor prognosis and enhances EGF-induced malignant phenotypes via activation of the EGFR–STAT3 pathway in endometrial cancer. Gynecol Oncol (2013) 128(3):560–7. 10.1016/j.ygyno.2012.12.012 23262208

[28] Li Q Jin W Jin Y Zheng Z Zhou X Wang Q Clinical effect of MUC1 and its relevance to BRAF V600E mutation in papillary thyroid carcinoma: a case–control study. Cancer Manage Res (2018) 10:1351–8. 10.2147/CMAR.S161501 PMC5985787 29881305

[29] Nam KH Noh TW Chung SH Lee SH Lee MK Hong SW Expression of the membrane mucins MUC4 and MUC15, potential markers of malignancy and prognosis, in papillary thyroid carcinoma. Thyroid (2011) 21(7):745–50. 10.1089/thy.2010.0339 21615302

[30] Byeon HK Na HJ Yang YJ Kwon HJ Chang JW Ban MJ c-Met-mediated reactivation of PI3K/AKT signaling contributes to insensitivity of BRAF(V600E) mutant thyroid cancer to BRAF inhibition. Mol Carcinog (2016) 55(11):1678–87. 10.1002/mc.22418 26456083

[31] Hayes DN Lucas AS Tanvetyanon T Krzyzanowska MK Chung CH Murphy BA Phase II efficacy and pharmacogenomic study of Selumetinib (AZD6244; ARRY-142886) in iodine-131 refractory papillary thyroid carcinoma with or without follicular elements. Clin Cancer Res (2012) 18(7):2056–65. 10.1158/1078-0432.CCR-11-0563 PMC5157199 22241789

[32] Barollo S Bertazza L Baldini E Ulisse S Cavedon E Boscaro M The combination of RAF265, SB590885, ZSTK474 on thyroid cancer cell Lines deeply impact on proliferation and MET and PI3K/Akt signaling pathways. Invest New Drugs (2014) 32(4):626–35. 10.1007/s10637-014-0108-3 24821574

